# The effect of body position on pulmonary function: a systematic review

**DOI:** 10.1186/s12890-018-0723-4

**Published:** 2018-10-11

**Authors:** Shikma Katz, Nissim Arish, Ariel Rokach, Yacov Zaltzman, Esther-Lee Marcus

**Affiliations:** 1 Chronic Ventilator-Dependent Division, Herzog Medical Center, POB 3900, Jerusalem, Israel; 2 Pulmonary Institute, Shaare Zedek Medical Center, POB 3235, Jerusalem, Israel; 3 Recanati School for Community Health Professions, Faculty of Health Sciences, Ben Gurion University of the Negev, Beer Sheva, Israel; 4 Hebrew University-Hadassah Faculty of Medicine, Jerusalem, Israel

**Keywords:** Body position, Lung volume, Physical therapy, Positioning, Posture, Pulmonary function, Sitting, Supine, Standing

## Abstract

**Background:**

Pulmonary function tests (PFTs) are routinely performed in the upright position due to measurement devices and patient comfort. This systematic review investigated the influence of body position on lung function in healthy persons and specific patient groups.

**Methods:**

A search to identify English-language papers published from 1/1998–12/2017 was conducted using MEDLINE and Google Scholar with key words: body position, lung function, lung mechanics, lung volume, position change, positioning, posture, pulmonary function testing, sitting, standing, supine, ventilation, and ventilatory change. Studies that were quasi-experimental, pre-post intervention; compared ≥2 positions, including sitting or standing; and assessed lung function in non-mechanically ventilated subjects aged ≥18 years were included. Primary outcome measures were forced expiratory volume in 1 s (FEV1), forced vital capacity (FVC, FEV1/FVC), vital capacity (VC), functional residual capacity (FRC), maximal expiratory pressure (PEmax), maximal inspiratory pressure (PImax), peak expiratory flow (PEF), total lung capacity (TLC), residual volume (RV), and diffusing capacity of the lungs for carbon monoxide (DLCO). Standing, sitting, supine, and right- and left-side lying positions were studied.

**Results:**

Forty-three studies met inclusion criteria. The study populations included healthy subjects (29 studies), lung disease (nine), heart disease (four), spinal cord injury (SCI, seven), neuromuscular diseases (three), and obesity (four). In most studies involving healthy subjects or patients with lung, heart, neuromuscular disease, or obesity, FEV1, FVC, FRC, PEmax, PImax, and/or PEF values were higher in more erect positions. For subjects with tetraplegic SCI, FVC and FEV1 were higher in supine vs. sitting. In healthy subjects, DLCO was higher in the supine vs. sitting, and in sitting vs. side-lying positions. In patients with chronic heart failure, the effect of position on DLCO varied.

**Conclusions:**

Body position influences the results of PFTs, but the optimal position and magnitude of the benefit varies between study populations. PFTs are routinely performed in the sitting position. We recommend the supine position should be considered in addition to sitting for PFTs in patients with SCI and neuromuscular disease. When treating patients with heart, lung, SCI, neuromuscular disease, or obesity, one should take into consideration that pulmonary physiology and function are influenced by body position.

**Electronic supplementary material:**

The online version of this article (10.1186/s12890-018-0723-4) contains supplementary material, which is available to authorized users.

## Background

Pulmonary function tests (PFTs) provide objective, quantifiable measures of lung function. They are used to evaluate and monitor diseases that affect heart and lung function, to monitor the effects of environmental, occupational, and drug exposures, to assess risks of surgery, and to assist in evaluations performed before employment or for insurance purposes. Spirometric examination is the most common form of PFT [1]. According to ATS/ERS guidelines, PFTs may be performed either in the sitting or standing position, and the position should be recorded on the report. Sitting is preferable for safety reasons to avoid falling due to syncope [2], and might also be more convenient because of the measurement devices and patient comfort. However, people who suffer from neuromuscular disease, morbid obesity, and other conditions may find it difficult to sit or stand during this test, which may influence their results.

One of the main goals of positioning, and specifically the use of upright positions, is to improve lung function in patients with respiratory disorders, heart failure, neuromuscular disease, spinal cord injury (SCI), and obesity, and in the past 20 years, various studies regarding the influence of body position on respiratory mechanics and/or function have been published. However, we did not find a systematic review that integrates findings from studies involving non-mechanically ventilated adults to derive clinical implications for respiratory care and pulmonary function test (PFT) execution.

We aimed to systematically review studies that evaluated the effect of body position on lung function in healthy subjects and non-mechanically ventilated patients with lung disease, heart disease, SCI, neuromuscular disease, and obesity.

## Methods

**Fig. 1:**
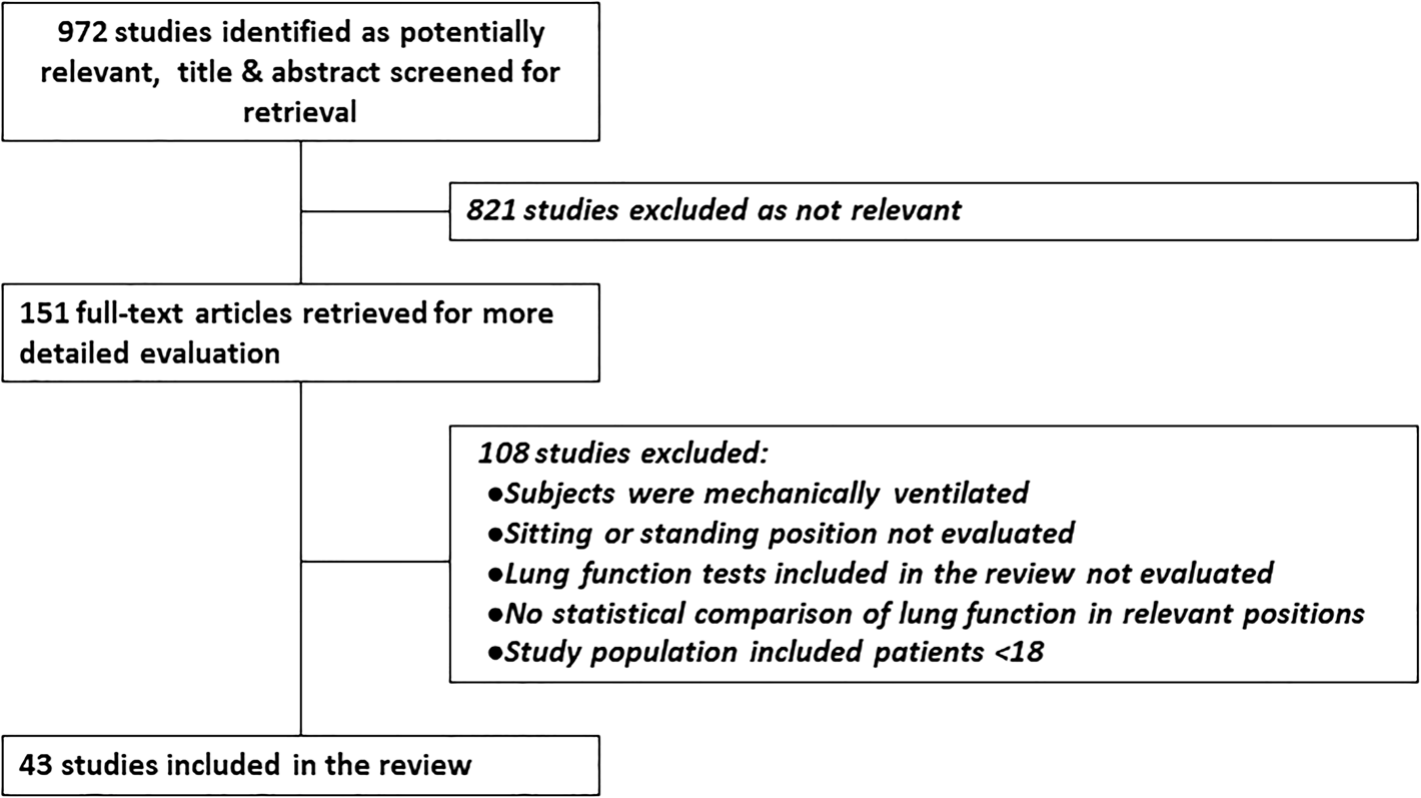
Study flow diagram

Two researchers (SK., E-LM.) searched MEDLINE and Google Scholar for studies published from January 1998–December 2017 using the key words body position, lung function, lung mechanics, lung volumes, position change, positioning, posture, PFTs, sitting, standing, supine, ventilation, and ventilatory change, in various combinations. Each search term combination included at least one key word related to pulmonary function and at least one related to body position. The year 1998 was chosen as the beginning point due to the publication of the seminal study by Meysman and Vincken [3]. A total of 972 abstracts identified in the search were screened by the same two researchers, and full text of 151 potentially relevant articles was obtained. The full texts were evaluated and categorized, and 108 articles not fulfilling the inclusion criteria were excluded (Fig. 1).

Articles were included if they met the following criteria: (1) Quasi-experimental, pre-post intervention. (2) Two or more body positions compared, including at least the sitting or standing position. (3) Outcome measures included assessment of lung function by forced vital capacity (FVC), forced expiratory volume in 1 s (FEV1), FEV1/FVC, vital capacity (VC), functional residual capacity (FRC), maximal expiratory pressure (PEmax), maximal inspiratory pressure (PImax), peak expiratory flow (PEF), total lung capacity (TLC), residual volume (RV), or diffusing capacity of the lungs for carbon monoxide (DLCO). (4) Study population of non-mechanically ventilated subjects. (5) Participants aged ≥18 years. (6) English language. Studies assessing lung function using other criteria and those without statistical comparisons of lung function in different positions, those enrolling individuals < 18 years or on mechanical ventilation, published conference abstracts, and systematic reviews were excluded.

### Positions studied

1. Standing – unsupported active standing
2. Sitting – sitting on a chair or wheelchair with the backrest at 90° and all limbs supported
3. Supine – lying flat on the back
4. Right-side lying (RSL) – lying straight on the right side
5. Left-side lying (LSL) – lying straight on the left side

### Outcome measures and defined thresholds for clinical significance

1. FVC – forced vital capacity
  Change of 200 ml or 12% from baseline values in FVC [4]
2. FEV1– forced expiratory volume in 1 s
  Change of 200 ml or 12% from baseline values in FEV1 [4]
3. FEV1/FVC – forced expiratory volume in 1 s divided by forced vital capacity
  FEV1/FVC < 0.7 is defined as obstructive disease
4. VC – vital capacity
5. FRC – functional residual capacity
  Change > 10% [5]
6. TLC – total lung capacity
  Change > 10% [5]
7. RV – residual volume
8. Maximal expiratory pressure (PEmax)
  Change ≥24 cmH2O [6–8]
9. Maximal inspiratory pressure (PImax)
  Change ≤ − 13 cmH2O [6–8]
10. Peak expiratory flow (PEF)
  Change > 10% or 60 L/min [9, 10]
11. Diffusing capacity of the lungs for carbon monoxide (DLCO)
  Change ≥10% in DLCO [11, 12]

Two experienced pulmonologists (NA, AR) reviewed the included studies in consensus to identify statistically significant and clinically important differences in pulmonary function. Results from articles included in the review were evaluated by all authors and categorized by study population, body positions studied, and outcome measures. Data from included studies was extracted by four authors (NA, AR, SK, E-LM.) independently and in consultation when questions arose. The review was performed according to the PRISMA guidelines [13].

Although these are not interventional studies, strictly speaking, we have chosen to assess them as “before and after intervention,” wherein the posture/position change is the maneuver of interest. Level of evidence was assessed according to the American Academy of Neurology (AAN) Classification of Evidence for therapeutic intervention [14]. Risk of bias was assessed according to the Quality Assessment Tool for Before-After (Pre-Post) Studies with No Control Group developed by the National Heart, Lung and Blood Institute (NHLBI) of the US National Institutes of Health (NIH) [15]. This tool is comprised of 12 questions assessing various aspects of the quality of the study. Two authors (E-LM, SK) independently scored each study using the technique from Kunstler et al. [16]. Differences were resolved in consensus, in consultation with a third author (YZ). The risk of bias was categorized as low (score 76–100%), moderate (26–75%) or high (0–25%).

## Results

### Studies included in the review

A total of 43 studies fully met inclusion criteria and were included in the review (Fig. 1). All studies used either consecutive, convenience, or volunteer sampling to enroll healthy individuals or subjects with various medical conditions. All studies provide Class III level of evidence.

The protocols and level of bias in the various studies are shown in Table 1 and Additional file 1: Table S1. Risk of bias was assessed as moderate in 41 studies and low in two. Quality issues were primarily related to sampling techniques for enrolling study participants. All studies used non-random sampling. Some studies investigating healthy subjects included convenience samples of young participants, mainly students. Only 7/43 studies reported sample size calculations required to reach statistical power. In addition, the details of the intervention protocol were not clearly reported in some studies (Table 1) and due to the nature of the study assessors could not be blinded to patient position or outcomes from previous tests.

**Table 1:** Study protocols

1st Author (year)	Procedure	Posture and Test Randomization	Adjustment period to posture prior to measurement	Risk of Bias
Antunes (2016) [45]	Mini Wright® (Clement Clarke International Ltd. Edinburgh Way Harlow, Essex, UK) peak flow meter portable device with a disposable mouthpiece	Random position order	1 min	Moderate
Badr (2002) [46]	Pressure manometer, vitalograph (Compact, Vitalograph Ltd., Buckingham, UK)	Random position order Random test order (PEF and PEmax) Subjects instructed on equipment use, practiced before test	5 min	Low
Baydur (2001) [35]	Spirometry	Random position order	N/A	Moderate
Ben-Dov (2009) [17]	Spirometry	N/A	N/A	Moderate
Benedik (2009) [52]	Helium dilution	First position always sitting	5 min	Moderate
Ceridon (2011) [18]	Spirometry, DLCO measured by rebreathe technique	N/A	30 min supine position prior to test Time prior to seated measurement not mentioned	Moderate
Chang (2005) [53]	Spirometry, FRC measured using helium dilution	First position always supine	5 min	Moderate
Costa (2015) [54]	Mouth pressure meter	Random position order	10 min	Moderate
De (2012) [29]	Spirometry	First position was always sitting	N/A	Moderate
Elkins (2005) [47]	Pressure manometer, spirometry - mass flow sensor	Random position and test order (PEF, PEmax) Subjects instructed on equipment use, practiced before test	5 min	Low
Faggiano (1998) [58]	Single breath technique using a Medical Graphics PF/DX module (Medical Graphics St. Paul, Minn, USA) for determining DLCO	Random position order	10 min	Moderate
Ganapathi (2015) [19]	Digital spirometry (BIOPAC System Inc. Goleta, California, USA)	N/A	N/A	Moderate
Gianinis (2013) [48]	Portable peak expiratory flow-device	Random position order	N/A	Moderate
Kim (2012) [36]	Spirometry	N/A	N/A	Moderate
Linn (2000) [33]	Spirometry	Random position order	N/A	Moderate
Manning (1999) [20]	Spirometry, single breath for determining DLCO	Two protocols (Session A & B). First chosen at random then alternated for successive subjects. First position always sitting.	15 min	Moderate
McCoy (2010) [49]	Peak flow meter	Random position order. Subjects instructed on equipment use, practiced before test	N/A	Moderate
Melam (2014) [30]	Spirometry (Excel/PC-based pulmonary function tests)	Random position order	N/A	Moderate
Meysman (1998) [3]	Spirometry, peak flow meter	Random position order	10 min	Moderate
Miccinilli (2016) [40]	Spirometry	N/A	N/A	Moderate
Mohammed (2017) [31]	Spirometry	Order of positions always standing, sitting, supine, lateral decubitus	N/A	Moderate
Myint (2017) [42]	Spirometry	Order of positions was standing, sitting, supine	N/A	Moderate
Naitoh (2014) [39]	Spirometry, breath dynamometer (Chest Co. Ltd)	First position always sitting	N/A	Moderate
Ogiwara (2002) [55]	Vitalpower KH-101 (Chest M.I. Inc., Japan)	Random position order	10 min	Moderate
Ottaviano (2016) [50]	Peak flow meter	Random position order	N/A	Moderate
Palermo (2005) [21]	Spirometry, DLCO measured by a single breath technique	Random position order	15 min	Moderate
Park (2010) [34]	Spirometry	N/A	N/A	Moderate
Patel (2015) [22]	Spirometry	First position always sitting	N/A	Moderate
Peces-Barba (2004) [56]	Single breath technique, rebreathing technique for determining DLCO	N/A	3–5 min	Moderate
Poussel (2014) [38]	Spirometry	Random position order	N/A	Moderate
Razi (2007) [32]	Spirometry	Alternately sitting, standing	N/A 15 min between positions	Moderate
Roychowdhury (2011) [44]	Spirometry	N/A	N/A 5 min rest between positions	Moderate
Saxena (2006) [23]	Spirometry	N/A	N/A	Moderate
Sebbane (2015) [41]	Spirometry, multiple breath helium dilution method	First position always sitting	N/A	Moderate
Stewart (2000) [24]	Single breath method for determining DLCO	N/A, 72 h between positions	15 min	Moderate
Terson de Paleville (2014) [37]	Spirometry, MP45–36-350 differential pressure transducer Validyne Engineering, (Northridge Ca, USA)	First position always sitting	30 min	Moderate
Terzano (2009) [57]	Single breath DLCO technique	Random position order	At least 15 min	Moderate
Tsubaki (2009) [28]	Micro RPM 01 (Micro Medical, UK), spirometry	Random position order	N/A	Moderate
Varrato (2001) [25]	Spirometry	N/A	N/A	Moderate
Vilke (2000) [26]	Spirometry	First position always supine/prone	N/A	Moderate
Wallace (2013) [51]	Peak flow meter	Random position order	N/A	Moderate
Watson (2005) [43]	Multi-breath helium dilution, spirometry	N/A	N/A	Moderate
Yap (2000) [27]	Spirometry, FRC was measured using helium dilution	First position always sitting	5 min	Moderate

Risk of bias was assessed using the Quality Assessment Tool for Before-After (Pre-Post) Studies with No Control Group [15, 16]

*DLCO* Diffusing capacity of the lungs for carbon monoxide, *FRC* Functional residual capacity

*N/A* Not available, not reported in the study

A summary of study characteristics, including the positions studied, outcome measures, and main results according to the study population, is shown in Table 2. Out of 43 studies, 29 included healthy subjects, nine included patients with lung disease, four included patients with heart disease, seven included patients with SCI, three included patients with neuromuscular diseases, and four included patients with obesity. Additional file 2: Table S2 summarizes only the statistically significant findings for each relevant outcome variable, according to position, for each of the populations studied.

**Table 2:** Summary of study characteristics according to study population

1st Author (Year)	No of Participants	Age (Years)	Population	Positions	Pulmonary Function	Main Findings
Meysman (1998) [3]	31	25.6 ± 3.8	Healthy	Sitting Supine RSL LSL	PEF, FVC, FEV1, PImax, PEmax	FVC, FEV1, PEF: sitting>supine LSL, RSL, PImax: sitting>supine PEmax: *p* > 0.05 between positions
Manning (1999) [20]	19	62.8 ± 6.8	Healthy older adults	Sitting LSL RSL	FVC, FEV1 DLCO/VA	FVC, FEV1: sitting>RSL & LSL DLCO/VA: *p* > 0.05 between positions
Vilke (2000) [26]	20	Range 18–50	Healthy males	Sitting Supine	FVC, FEV1	FVC, FEV1: sitting>supine
Stewart (2000) [24]	10	22.3 ± 2.4	Healthy males	Sitting Supine	FVC, VC, FEV1, FEV1/FVC, PEF, DLCO	FVC, PEF: sitting>supine DLCO: sitting< supine FEV1, VC, FEV1/FVC: *p* > 0.05 between positions
Yap (2000) [27]	10	62.2±1.2 *Mean±SE*	Healthy	Sitting Supine	FVC, FEV1, FEV1/FVC, FRC	FVC, FEV1, FRC: sitting>supine FEV1/FVC: *p* > 0.05 between positions
Varrato (2001) [25]	15	Mean 41	Healthy	Sitting Supine	FVC	FVC: sitting>supine
Badr (2002) [46]	25	34.0 ± 14.9	Healthy	Standing Sitting Supine RSL	PEmax, PEF	PEmax: standing>other positions PEmax: sitting>supine & RSL PEF: standing>other positions
Ogiwara (2002) [55]	20	Mean 22.8±2.1 Range 21–28	Healthy	Sitting Supine RSL LSL	PEmax, PImax	PEmax, PImax: *p* > 0.05 between positions
Peces-Barba (2004) [56]	14	37.5±11.5	Healthy	Sitting Supine	DLCO	DLCO: sitting<supine
Chang (2005) [53]	20	28.3 ± 4.8	Healthy males	Standing Supine	FRC	FRC: standing>supine
Palermo (2005) [21]	14	61 ± 8	Healthy	Sitting Supine LSL RSL	FEV1, FVC, VC, DLCO	FEV1, FVC, VC: *p* > 0.05 between positions DLCO: sitting>LSL & RSL
Watson (2005) [43]	5	Mean 57	Healthy	Sitting Supine	TLC, VC, RV, FRC	TLC, VC, RV: *p* > 0.05 between positions FRC sitting>supine
Saxena (2006) [23]	80	Males 21.3 ± 1.5 Females 19.6 ± 1.3	Healthy	Standing Sitting Supine	FEV1, FVC, FEV1/FVC, PEF	FEV1, FVC, PEF: standing>supine FEV1/FVC: sitting>supine
Ben-Dov (2009) [17]	7	44 ± 10	Healthy	Sitting Supine	FVC	FVC: *p* > 0.05 between positions
Terzano (2009) [57]	10	59.0 ± 9.3	Healthy	Standing Sitting Supine	DLCO	DLCO: *p* > 0.05 between positions
Tsubaki (2009) [28]	15	22.7 ± 2.3	Healthy females	Sitting Supine	FVC, FEV1, FEV1/FVC, VC, PImax, PEmax	FEV1/FVC: sitting>supine FVC, FEV1, VC, PImax, PEmax: *p* > 0.05 between positions
McCoy (2010) [49]	182	23.5 ± 2.5 (healthy and asthmatic patients)	Healthy	Standing, Sitting	PEF	PEF: *p* > 0.05 between positions
Ceridon (2011) [18]	12	63 ± 9	Healthy	Sitting Supine	FVC, FEV1, FEV1/FVC, DLCO	FVC, FEV1, DLCO: sitting>supine FEV1/FVC: *p* > 0.05 between positions
Roychowdhury (2011) [44]	100	Range 19–22	Healthy	Sitting Supine	VC	VC: supine>sitting in females VC: *p* > 0.05 between positions in males
Gianinis (2013) [48]	30	22.2 ± 2.4	Healthy	Sitting Supine RSL LSL	PEF	PEF: sitting>supine & RSL
Wallace (2013) [51]	94	23.9 ± 3.7	Healthy	Standing Sitting	PEF	PEF: standing>sitting
Naitoh (2014) [39]	20	28 ± 1.4	Healthy	Sitting Supine	FEV1,VC PEmax, PImax	FEV1, VC: sitting>supine PEmax, PImax: *p* > 0.05 between positions
Costa (2015) [54]	63	19.7 ± 1.5	Healthy	Sitting Supine	PImax, PEmax	PImax, PEmax: sitting>supine
Ganapathi (2015) [19]	20	Range 18–25	Healthy	Standing Sitting Supine RSL LSL	FVC, FEV1, FEV1/FVC	FVC, FEV1, FEV1/FVC: standing>supine, RSL& LSL FEV1: standing>sitting sitting>RSL FEV1/FVC: sitting>LSL
Patel (2015) [22]	45	Median 21 Range 19–23	Healthy	Standing, Sitting Supine	FVC, FEV1, PEF	FVC, FEV1, PEF: sitting>standing FVC, FEV1, PEF: sitting>supine
Antunes (2016) [45]	30	22.7 ± 2.4	Healthy	Sitting Supine	PEF	PEF: sitting>supine
Miccinilli (2016) [40]	20	33.6 ± 10.5	Healthy	Sitting Supine	VC, FEV1	VC, FEV1: *p* > 0.05 between positions
Ottaviano (2016) [50]	76	40 ± 16	Healthy	Standing Sitting	PEF	PEF: standing>sitting
Myint (2017) [42]	15	22.6 ± 2.0	Healthy	Standing, Sitting Supine	FEV1/FVC	FEV1/FVC: *p* > 0.05 between positions
Badr (2002) [46]	11	66.8 ± 12.6	Chronic airflow limitation	Standing Sitting Supine RSL	PEmax, PEF	PEmax: standing>supine & RSL, PEmax: sitting>supine & RSL PEF: standing>sitting, supine, RSL
Elkins (2005) [47]	20	29 ± 8	Adult cystic fibrosis	Standing Sitting Supine RSL	PEmax, PEF	PEmax: standing & sitting>RSL PEF: standing>supine & RSL
Razi (2007) [32]	49	42.6 ± 11.8	Obesity, asthma *(Mean BMI 36±5)*	Standing Sitting	FVC, FEV1, FEV1/FVC	FVC, FEV1, FEV1/FVC: *p* > 0.05 between positions
Terzano (2009) [57]	30 *Mild 10* *Moderate-severe 10* *Very severe 10*	Mild 57.3 ± 8.6 Moderate-severe 59.8 ± 9.1 Very severe 63.7 ± 5.5	COPD	Standing Sitting Supine	DLCO	DLCO: *p* > 0.05 between positions
McCoy (2010) [49]	29	23.5 ± 2.5 (Healthy and asthmatic patients)	Asthma	Standing Sitting	PEF	PEF: *p* > 0.05 between positions
De (2012) [29]	75	61.2 ± 9.2	COPD	Standing Sitting	FVC, FEV1	FVC, FEV1: *p* > 0.05 between positions
Melam (2014) [30]	30	34.3 ± 3.7	Asthma	Standing, Sitting Supine RSL LSL	FVC, FEV1	FVC, FEV1: standing>supine
Mohammed (2017) [31]	20	39.2 ± 8.0	Asthma	Standing Sitting Supine RSL LSL	FVC, FEV1, PEF	FVC, FEV1, PEF: standing>supine, RSL, LSL FVC, FEV1: standing>sitting
Myint (2017) [42]	15	22.3 ± 2.0	Asthma	Standing Sitting Supine	FEV1/FVC	FEV1/FVC: *p* > 0.05 between positions
Faggiano (1998) [58]	32	59 ± 10	CHF, males	Sitting Supine	DLCO	DLCO: *p* > 0.05 between positions ↓DLCO in sitting in a subgroup of patients with decrease in mean pulmonary arterial pressure in this position ↑DLCO in sitting in a subgroup of patients with increase in mean pulmonary arterial pressure in this position
Yap (2000) [27]	10	61.4 ± 2.0 * Mean±SE*	CHF	Sitting Supine	FEV1, FVC, FEV1/FVC, VC, FRC	FVC, FEV1 VC: sitting>supine FEV1/FVC, FRC: *p* > 0.05 between positions
Palermo (2005) [21]	14	62 ± 8	CHF	Sitting Supine LSL RSL	FEV1, FVC, VC, DLCO	FEV1, FVC: sitting>RSL & LSL DLCO: sitting>RSL &LSL VC: *p* > 0.05 between positions
Ceridon (2011) [18]	24	65 ± 8	CHF	Sitting Supine	FEV1, FVC, FEV1/FVC, DLCO	FEV1, FVC: sitting>supine FEV1/FVC: *p* > 0.05 between positions DLCO: *p* > 0.05 between positions
Linn (2000) [33]	222 *Tetraplegia 98* *Paraplegia 124*	40 ± 11	SCI	Sitting Supine	FVC, FEV1, PEF	FVC, FEV1, PEF: sitting<supine in complete tetraplegia FVC, FEV1, PEF: *p* > 0.05 between positions in paraplegia
Baydur (2001) [35]	74 *C3–7 injury tetraplegia 31 T—L4 injury paraplegia 43*	40 ± 12	SCI	Sitting Supine	FVC, FEV1	FVC, FEV1: *p* > 0.05 between positions
Ben Dov (2009) [17]	12	42 ± 11	Neurologically stable *C5–8 tetraplegia*	Sitting Supine	FVC	FVC: sitting<supine
Park (2010) [34]	43	35.0 ± 12.6	SCI *C6-C8*	Sitting Supine	FVC	FVC: sitting<supine
Kim (2012) [36]	45 *Cervical 15* *Thoracic 13* *Lumbar 17*	Cervical 43.2 ± 1.3 Thoracic 49.8 ± 4.9 Lumbar 52.2 ± 4.4	SCI	Sitting Supine	FVC, FEV1	FVC, FEV1: *p* > 0.05 between positions within cervical/thoracic/lumbar subgroups FVC, FEV1: sitting<supine in cervical and thoracic injury FVC, FEV1: sitting>supine in lumbar injury Statistically significant difference in the effect of position between cervical/thoracic/lumbar subgroups
Terson de Paleville (2014) [37]	27 *Complete motor injury 13 Incomplete motor injury 14 Cervical 15, Thoracic 12*	40 ± 14	SCI	Sitting Supine	FVC, FEV1, PEmax, PImax	FVC, FEV1: *p* > 0.05 between positions for all patients together FVC: sitting<supine in cervical/complete motor injury FVC: sitting>supine in thoracic incomplete motor injury FEV1 sitting> supine in incomplete motor injury FEV1: sitting>supine in thoracic incomplete motor injury PEmax: sitting>supine all patients PEmax: sitting>supine in complete motor injury PEmax: sitting>supine in cervical incomplete motor injury PImax: *p* > 0.05 between positions for all patients together PImax: sitting>supine in thoracic complete motor injury
Miccinilli (2016) [40]	20 *C3–7 tetraplegia 9; T1–8 paraplegia 11*	Tetraplegia 29.4 ± 10.5 Paraplegia 36.6 ± 10.3	SCI	Sitting Supine	VC, FEV1	VC, FEV1: sitting<supine
Varrato (2001) [25]	38	61	ALS	Sitting Supine	FVC	FVC: sitting>supine
Park (2010) [34]	45	54.4 ± 11.1	ALS	Sitting Supine	FVC	FVC: sitting>supine
Poussel (2014) [38]	58	42.6 ± 12.9	Myotonic dystrophy	Sitting Supine	FVC, FEV1	FVC, FEV1: sitting>supine
Watson (2005) [43]	10	49 ± 6 *Mean ± SE*	Obesity Mean BMI 44±3 *Mean±SE*	Sitting Supine	TLC, VC, RV, FRC,	TLC, VC, RV, FRC: *p* > 0.05 between positions
Razi (2007) [32]	51	39.86 ± 10.1	Obesity *Mean BMI 36.7±4.1*	Standing Sitting	FVC, FEV1, FEV1/FVC	FVC, FEV1, FEV1/FVC: *p* > 0.05 between positions
Benedik (2009) [52]	32	Range 18–75	Healthy, mild-moderate obesity *Mean BMI 32.7±3.5*	Sitting Supine	FRC	FRC: sitting>supine
Sebbane (2015) [41]	12	44 ± 14	Morbid obesity *Mean BMI 45±5* S/P bariatric surgery *Mean BMI 31 ± 5*	Sitting Supine	TLC, RV, VC, FRC, FEV1	FEV1: sitting>supine in morbid obesity TLC, RV, FRC, VC: *p* > 0.05 between positions in morbid obesity FRC, FEV1: sitting>supine in s/p bariatric surgery TLC, RV, VC: *p* > 0.05 between positions in s/p bariatric surgery

Values are mean ± S.D. unless specified other

*ALS* Amyotrophic lateral sclerosis, *BMI* Body mass index, *CHF* Congestive heart failure, *COPD* Chronic obstructive pulmonary disease, *DLCO* Diffusing capacity of the lungs for carbon monoxide, *DLCO/VA* Diffusing capacity of the lung for carbon monoxide divided by alveolar volume, *FEV1* Forced expiratory flow in 1 s, *FRC* Functional residual capacity, *FVC* Forced vital capacity, *LSL* Left side lying, *PEF* Peak expiratory flow, *PEmax* Maximal expiratory pressure, *PImax* Maximal inspiratory pressure, *RSL* Right side lying, *RV* Residual volume, *SCI* Spinal cord injury, *S/P* Status post, *TLC* Total lung capacity, *VC* Vital capacity

### FVC

The association between FVC and body position in healthy subjects was investigated in 13 studies [3, 17–28]. There was a clinical and statistically significant increase in FVC in sitting vs. supine positions [3, 18, 22–27], in sitting vs. RSL and LSL [3, 21], standing vs. supine [19, 23], and standing vs. RSL and LSL [19]. In a smaller number of studies there was no change between standing and sitting [19], sitting and supine [17, 21, 28] or sitting and RSL or LSL [21], and one study [22] found a decrease in FVC from sitting to standing that was statistically but not clinically significant. Thus, in the majority of studies the more upright position was associated with increased FVC.

Four studies included subjects with lung disease [29–32]. Among asthmatic patients in one study FVC increased significantly from supine to standing [30]; however, there was no significant difference between standing and sitting or between sitting and supine, RSL, or LSL. Another study reported a statistically and clinically significant increase in FVC in standing vs. sitting, supine, RSL, and LSL and in sitting vs. supine, RSL and LSL [31]. Among obese asthmatic patients [32], and among patients with chronic obstructive pulmonary disease (COPD) [29], no difference was found in FVC between standing and sitting.

Three studies included subjects with congestive heart failure (CHF) [18, 21, 27]. In one study, FVC was reported 200 ml higher in sitting vs. RSL and LSL [21], and in the other two studies FVC was higher in sitting vs. supine by 350–400 ml, which has clinical significance [18, 27].

Six studies included patients with SCI [17, 33–37]. The effect of body position on FVC depends on the level and extent of injury. Among those with cervical SCI, FVC was higher in the supine vs. sitting position [17, 33, 34]. Other studies [35–37] did not find significant differences in FVC for patients with SCI in a pooled group of all levels of injury for these positions. However, in patients with cervical SCI, as well as those with thoracic injury in one study [36], there was an increased FVC in the supine vs. sitting, while in those with thoracic or lumbar injury FVC was higher in the sitting position [37]. The differences did not always reach statistical significance. Nevertheless, it is important to note that in these debilitated patients with SCI, even a small change in FVC is probably clinically significant.

Three studies evaluated patients with neuromuscular diseases [25, 34, 38]. In patients with myotonic dystrophy and in those with amyotrophic lateral sclerosis (ALS), there was a clinically and statistically significant decrease in FVC from sitting to supine [25, 34, 38]. In subjects with obesity (mean BMI 36.7) no significant difference was reported between standing and sitting [32].

### FEV1

In healthy subjects, FEV1 was reported to be higher in sitting vs. supine [3, 18, 22, 23, 26, 27, 39], in sitting vs. RSL and LSL [3, 19, 20], in standing vs. sitting [23], and in standing vs. sitting, supine, RSL, and LSL [19]. However, other studies [21, 24, 28, 40] did not find significant difference for FEV1 between sitting and supine, RSL, and LSL. One study [22] reported a decrease of 120 ml in FEV1 from sitting to standing, which is statistically but not clinically significant.

Among asthmatic patients, FEV1 was higher in the standing vs. supine position, a statistically and clinically significant change; however, there was no significant difference between sitting vs. supine, RSL, and LSL positions [30]. Another study in asthmatic patients reported FEV1 to be higher in standing vs. sitting, supine, RSL, and LSL, and in sitting  vs. supine, RSL and LSL [31]. Among obese asthmatic patients and those with COPD, there was no significant difference in FEV1 between standing and sitting [29, 32].

In subjects with CHF, one study found a statistically and clinically significant increase in FEV1 in sitting vs. RSL and LSL, but no difference between sitting and supine [21], while two other studies reported higher FEV1 in sitting vs. supine [18, 27].

In patients with SCI, FEV1 was recently reported to increase from sitting to supine [40]; however, other studies found that the effect of position on FEV1 in those with SCI depends on the level and extent of injury. In one study among all subjects with SCI, FEV1 was not significantly influenced by moving from sitting to supine [35], but patients with cervical injuries showed a tendency for increased FEV1 in the supine vs. sitting position while those with thoracic injuries tended towards increased FEV1 in the sitting position. Along the same vein, another study [36] found an increase is FEV1 in the sitting vs. the supine position in patients with lumbar injury while FEV1 was higher in the supine position for those with cervical spine or thoracic injuries. Although the differences between positions were not statistically significant, the effect of level of injury was statistically and clinically significant.

In another study [33], FEV1 was higher in supine vs. sitting in patients with complete tetraplegia, while in patients with incomplete injury there was no significant difference between positions. Another group [37] reported no significant change in FEV1 between the sitting and supine positions for a pooled group of patients with SCI, but in the subgroup of patients with incomplete motor injury and in those with incomplete thoracic motor injury there was a decrease in the supine position.

In patients with myotonic dystrophy, FEV1 decreased from sitting to supine [38]. Among those with obesity, FEV1 was higher in sitting vs. supine both before and after bariatric surgery [41]. In another study among obese patients, there was no difference in FEV1 between standing and sitting [32].

### FEV1/FVC

Seven studies compared FEV1/FVC for different body positions in healthy subjects [18, 19, 23, 24, 27, 28, 42]. In several studies, FEV1/FVC was reported to be higher in sitting vs. supine [23, 28], in sitting vs. LSL [19], and in standing vs. supine, RSL, and LSL [19]; however, FEV1/FVC was > 70% in all body positions so the difference was not clinically significant. Other studies found no difference between sitting and supine [18, 24, 27] or standing, sitting, and supine [42].

Among subjects with asthma, CHF, and obesity no statistically significant difference in FEV1/FVC was found between the different body postures [18, 27, 32, 42].

### Vital capacity

The effect of body position on vital capacity was evaluated in six studies of healthy subjects [21, 24, 28, 39, 43, 44]. In most studies no difference was reported between sitting and supine [21, 24, 28, 43] or between sitting and RSL or LSL [21]. One study [39] found that VC was higher in the sitting vs. supine position. However, another study [44] found that VC was higher in the supine vs. sitting position, but only in females.

In patients with CHF, VC was reported to be higher in sitting vs. supine in one study [27] while another study found no statistically significant difference between these positions [21]. In patients with spinal cord injury, VC was higher in the supine vs. sitting position [40]. In subjects with obesity, no difference in VC was reported between the sitting and supine positions [41, 43].

### PEF

PEF in different body positions was evaluated in 13 studies [3, 22–24, 31, 33, 45–51]. Eight studies evaluated only healthy adults [3, 22–24, 45, 48, 50, 51], three evaluated healthy subjects and patients with COPD or asthma [31, 46, 49], one included adult cystic fibrosis patients [47], and one included subjects with SCI [33]. Nine studies that compared standing or sitting positions vs. supine or RSL and LSL found higher PEF in standing and sitting [3, 22–24, 31, 45–48]. Three of six studies comparing the standing and sitting positions found higher PEF in standing [46, 50, 51] and one reported higher PEF in sitting [22]. However, it is most likely that none of the differences reported in PEF are clinically significant. In SCI patients with complete tetraplegia PEF was found to be 12% higher in the supine vs. sitting position [33].

### FRC

FRC was evaluated using helium dilution in five studies [27, 41, 43, 52, 53]. Among healthy subjects, FRC was higher in standing [53] and in sitting [27, 43] vs. supine, with the differences reaching statistical and clinical significance. However, the difference in sitting vs. supine was not significant among patients with obesity (mean BMI 44–45) [41, 43] or CHF [27], and was higher in sitting vs. supine in patients after bariatric surgery (mean BMI 31) [41]. Another study [52] involving subjects with mild-to-moderate obesity (mean BMI 32), reported that FRC was significantly higher both statistically and clinically in sitting vs. supine.

### Total lung capacity

Two studies that evaluated TLC using helium dilution in healthy subjects [43] and in subjects with obesity [41, 43] found no statistically significant difference between the sitting and supine positions.

### Residual volume

Two studies that evaluated RV using helium dilution in healthy subjects [43] and those with obesity [41, 43] found no statistically significant difference between sitting and supine.

### PEmax

Six studies investigated the association between body position and PEmax in healthy subjects [3, 28, 39, 46, 54, 55]. PEmax was higher in standing vs. supine, in standing vs. sitting and RSL, in sitting vs. supine [54], and in sitting vs. supine and RSL [46]; however, the differences reported in those studies were not clinically significant. Other studies found no difference in PEmax between sitting and supine [28, 39], or between sitting, supine, RSL, and LSL [3, 55].

In COPD patients, PEmax was higher in standing or sitting vs. supine or RSL [46], and was higher in standing and sitting vs. RSL in patients with cystic fibrosis [47]. The differences were not clinically significant.

In subjects with SCI, PEmax was significantly higher in sitting vs. supine for all subjects, and for patients with motor complete injury or incomplete cervical motor injury [37].

### PImax

In healthy subjects, PImax was improved in sitting vs. supine in two studies [3, 54]. However, other studies found no difference in PImax in sitting vs. supine [28, 39, 55], or sitting vs. RSL and LSL [3, 55]. In subjects with chronic SCI, no significant change was seen in PImax between sitting and supine, with the exception of a subgroup of patients with complete thoracic motor paresis where there was statistically and clinically significant improvement in sitting [37].

### DLCO

Seven studies evaluated the effect of body position on diffusion capacity; six included healthy subjects [18, 20, 21, 24, 56, 57], three included patients with CHF [18, 21, 58], and one included COPD patients [57].

Among healthy subjects, two studies [24, 56] found statistically and clinically significant improvement in DLCO in supine vs. sitting and one [57] found a trend towards increased DLCO in supine vs. sitting, however this difference did not reach statistical significance. One study [18] found DLCO to be higher in the sitting vs. supine positions while another study found no difference in DLCO between these positions [21]. One study [21] reported higher DLCO in sitting vs. side lying while another study [20] found no difference between these positions. In COPD patients, no statistically significant change in DLCO was found between the sitting and the supine position [57].

Three studies investigated diffusion capacity in patients with CHF [18, 21, 58]. One study [58] found that postural changes from the supine to sitting positions induced different responses in diffusion capacity. In some patients diffusion capacity improved in the sitting position and others showed no change or a decline. On the average no statistically significant difference was found between the two positions. The authors attributed the difference in responses to variations in pulmonary circulation pressures. Another study [18] found no significant difference in diffusion capacity between the sitting and the supine positions. Side-lying was reported to reduce DLCO in comparison to sitting in the third study [21].

## Discussion

Most studies in this systematic review of 43 papers evaluating the effect of body position on pulmonary function found that pulmonary function improved with more erect posture in both healthy subjects and those with lung disease, heart disease, neuromuscular diseases, and obesity. In patients with SCI, the effect is more complex and depends on the severity and level of injury. In contrast, diffusion capacity, as assessed by DLCO, increases in the supine position in healthy subjects while the effect in CHF patients is thought to depend upon pulmonary circulation pressure.

Decreased FVC in more recumbent positions may reflect both increased thoracic blood volume due to gravitational facilitation of venous return, which is more important in patients with heart failure, as well as cephalic displacement of the diaphragm due to abdominal pressure in the recumbent positions, which is more important in obese subjects [59]. In side-lying positions, even though only the dependent hemi-diaphragm is displaced, the effect on FVC appears to be similar to that observed in a supine position [59]. Other factors that may contribute to lower FVC values in side-lying positions include increased airway resistance and decreased lung compliance secondary to anatomical differences between the left and right lungs, as well as shifting of the mediastinal structures [20].

FEV1 was also higher in erect positions. Recumbent positions limit expiratory volumes and flow, which may reflect an increase in airway resistance, a decrease in elastic recoil of the lung, or decreased mechanical advantage of forced expiration, presumably affecting the large airways [20]. In asthmatic patients the increase in FVC while standing might be due to the increased diameter of the airways in this position [30].

In patients with CHF the lungs are stiff and heavy, and the heart is large and heavy, increasing the negative effects of lung-heart interdependence [60]. As cardiac dimension increases, lung volume, mechanical function, and diffusion capacity decrease [61, 62]; thus, the heart weighs on the diaphragm while sitting and on one of the lungs while in a side-lying position. This influences the ability of the lungs to expand laterally but allows the diaphragm to descend and the lungs to expand inferiorly. In side-lying positions, the heart weighs on one lung, compressing both the airways and lung parenchyma, leading to a reduction in FEV1 and FVC due to airway compression [21]. Both elastic (reduced lung compliance) and resistive loads are simultaneously increased in the supine position in CHF patients [63].

Changes in FVC from the sitting to supine positions may reflect diaphragm strength/paralysis. FVC is thus an important clinical tool for assessment of diaphragmatic weakness in patients with neuromuscular diseases [64]. In patients with ALS, supine FVC is a test of diaphragmatic weakness [65] that predicts orthopnea [25] and prognosis for survival [66, 67]. The American Academy of Neurology has concluded that in ALS patients, supine FVC is probably more effective than erect FVC in detecting diaphragm weakness and correlates better with symptoms of hypoventilation [68].

In patients with cervical SCI (tetraplegia), FVC and FEV1 increase in the supine vs. sitting position. The diaphragm increases its inspiratory excursion in the supine position because its muscle fibers are longer at end expiration, and they operate at a more effective point of their length-tension curve [69–71]. This mechanism is especially important in patients for whom the diaphragm is the main muscle for breathing, since their intercostal and abdominal muscles are inactive due to SCI.

FRC was reported to increase in upright positions in healthy subjects [27, 43, 53] and in patients with mild-to-moderate obesity [41, 52]. Changing from a supine to an upright position increases FRC due to reduced pulmonary blood volume and the descent of the diaphragm. This may change the point in which tidal breathing occurs in the volume-pressure curve, which leads to increased lung compliance, and thus an identical pressure change would produce a greater inspired volume if there is no change in respiratory drive [53]. However, among patients with CHF, no difference in FRC between sitting and supine was reported [27]. In heart failure, reduction in lung compliance in the supine position might reduce the passive change in lung volume, but FRC may be sustained above relaxation volume by an adjustment in respiratory muscle or glottal activity [27]. Among patients with obesity the sitting FRC was less than in healthy subjects but there was no further decrease in the supine position [43].

PEF, PEmax, and PImax were found to increase in upright positions in healthy subjects [3, 23, 24, 46, 48, 50, 51] and in those with lung diseases [31, 46, 47]. This may be related to changes in lung volumes with positions.

Standing and sitting have been shown to lead to the highest lung volumes [72, 73]. At higher lung volumes the elastic recoil of the lungs and the chest wall is greater. In addition, the expiratory muscles are at a more optimal region of the length-tension curve and thus are capable of generating higher intrathoracic pressure, potentially generating higher expiratory pressures and pushing air through narrow airways at high speed, which results in higher PEmax, PEF, and FEV1. As lung volumes decrease, muscle length becomes less optimal, which results in lower PEmax in sitting, compared to the standing position, and even lower in more recumbent positions. The change in PEmax influences PEF [46].

When standing, gravity pulls the mediastinal and abdominal structures down, creating more space in the thoracic cavity, which allows further expansion of the lungs and greater lung volumes [74]. This, along with the decrease in compression on the lung bases, allows alveoli to recruit and increases lung compliance. The inspiratory muscles can expand even more, which allows the diaphragm to continue contracting downwards, thus increasing lung volumes [46].

Sitting often leads to the somewhat reduced lung volumes compared with standing. This can be explained by several mechanisms. First, in sitting, abdominal organs are higher, interfering with diaphragmatic motion, thus enabling smaller inspiration. Second, the abdominal muscles are in a less optimal point in the length-tension curve, since the combination of hip flexion and higher position of the abdominal contents exert upward pressure. Third, the back of the chair may limit thoracic expansion. These three factors explain a slightly lower PEmax and PEF in sitting vs. standing [46].

Diaphragmatic strength is negatively affected by the supine position, and intrathoracic blood volume is increased. These factors lead to decreased PEmax and PEF in the supine position [3].

In side-lying positions (RSL or LSL), when the bed is flat, the abdominal contents fall forward. The dependent hemi-diaphragm is stretched to a good length for tension generation, while the nondependent hemi-diaphragm is more flattened. Changes in lung volumes may thus balance themselves out due to a better diaphragmatic contraction but decreased space in the thorax [46].

The decreased PImax observed in the supine position could be related to diaphragm overload by abdominal content displacement during maximal inspiratory effort, which could offset improved diaphragm position on the length-tension curve. In addition, the length of all other inspiratory muscles may become less optimal in supine position [75].

In patients with cervical spinal cord injury and high tetraplegia, PEF was found to be higher in the supine vs. sitting position [33] corresponding to the increase in FVC and FEV1 in the supine position.

In healthy subjects, most studies showed an increase in DLCO in supine vs. sitting [24, 56, 57]. This improvement is attributed to the moderate increase in alveolar blood volume in the supine position due to recruitment of lung capillary bed on transition from upright to supine. Age may attenuate this increase [76]. This may explain why a study that included participants with a mean age of 61 [21] found no difference in DLCO between sitting and supine.

In side-lying positions, the heart weighs on one lung, compressing both airways and lung parenchyma, reducing alveolar blood volume, and causing ventilation/ perfusion mismatch. Those effects caused reduction of diffusion capacity in the side-lying positions [21].

In COPD patients, there was no change in DLCO between sitting and supine [57]. This might be related to reduced FVC and alveolar damage in these patients. These effects might have negative impact on diffusion capacity, opposing the positive effect of the increase in blood volume in the alveoli [57].

In patients with CHF, different patterns of the effect of posture on DLCO were observed [58]. The change in DLCO was probably related to the change in alveolar blood volume, most likely due to differences in pulmonary artery pressure and heart dimensions [58].

### Limitations of the study

There are a few limitations to this review. First, the level of evidence of the studies is relatively low. However, in this type of research, due to the nature of the populations studied and the interventions applied, it is impossible to perform a randomized control study. Second, most studies were performed on a small number of subjects and all studies used either consecutive, convenience, or volunteer sampling. The review included only adult subjects and it is therefore not possible to generalize the results to children and adolescents. Finally, research protocols varied between studies and detailed information about protocols were often missing. Patient cooperation during lung function testing strongly influences results. This may explain contradictory results obtained in some cases. Studies that included subjects older than 60 years did not mention the cognitive function of participants, a factor that may influence patient cooperation.

Further research in this field is needed, including studies designed to evaluate lung function in a larger number of healthy participants as well as in individuals with a variety of medical conditions. There is also a need to use a standardized protocol including randomization of postures and times between tests (e.g. for wash-out of inhaled gasses or redistribution of blood volume) in different positions to enable a better comparison of outcomes.

## Conclusions

When performing pulmonary function tests, body position plays a role in its influence over test results. As seen in this review, a change in body position may have varying implications depending on the patient populations. American Thoracic Society (ATS) guidelines [2] recommend performing PFTs in the sitting or standing position, but the sitting position is usually preferred. The norms of those functions according to gender and age were established from tests performed in this position. This review suggests that for most of the subjects this is the preferred position for the test; however, clinicians should consider performing PFTs in other positions in selected patients. In patients with SCI, testing also in the supine position may provide important information. In patients with neuromuscular disorders, performing PFTs in the supine position may help to assess diaphragmatic function.

Positioning plays an important role in maximizing respiratory function when treating patients with various problems and diseases and it is important to know the implications of each position on the respiratory system of a specific patient. Understanding the influence of body position can give healthcare professionals better knowledge of optimal positions for patients with different diseases.

## Additional files

Additional file 1: **Table S1.** Scoring for papers included in the systematic review based on the Quality Assessment Tool for Before-After (Pre-Post) Studies with No Control Group of the National Heart, Lung and Blood Institute [3, 15–31, 33–58]. (DOCX 63 kb)

Additional file 2: **Table S2.** Statistically significant differences in pulmonary function between the various body positions [3, 17–28, 30, 31, 33, 34, 37–41, 43–48, 50–54, 56]. (DOCX 104 kb)

## Abbreviations

AAN: American Academy of Neurology
ALS: Amyotrophic lateral sclerosis
ATS: American Thoracic Society
CHF: Congestive heart failure
COPD: Chronic obstructive pulmonary disease
DLCO: Diffusing capacity of the lungs for carbon monoxide
ERS: European Respiratory Society
FEV1: Forced expiratory volume in 1 s
FRC: Functional residual capacity
FVC: Forced vital capacity
LSL: Left side lying
PEF: Peak expiratory flow
PEmax: Maximal expiratory pressure
PFT: Pulmonary function test
PImax: Maximal inspiratory pressure
RSL: Right side lying
RV: Residual volume
SCI: Spinal cord injury
TLC: Total lung capacity
VC: Vital capacity
