# Supplementary material for: The effect of body position on pulmonary function: a systematic review
Source: BMC Pulm Med. 2018 Oct 11;18:159. doi: 10.1186/s12890-018-0723-4 (PMC6180369; doi:10.1186/s12890-018-0723-4)
Supplement: Supplementary file 1 — Table S1. Scoring for papers included in the systematic review based on the Quality Assessment Tool for Before-After (Pre-Post) Studies with No Control Group of the National Heart, Lung and Blood Institute [3, 15–31, 33–58]. (DOCX 63 kb) [file 12890_2018_723_MOESM1_ESM.docx]

**Additional file 1**

**Table S1: Scoring for papers included in the systematic review based on the Quality Assessment Tool for Before-After (Pre-Post) Studies with No Control Group of the National Heart, Lung and Blood Institute [15]**

| **Study, 1^st^ Author, Year** | ***Question Number^a^*** | | | | | | | | | | | | **Study Score (%) [16]** |
| --- | --- | --- | --- | --- | --- | --- | --- | --- | --- | --- | --- | --- | --- |
|  | **1** | **2** | **3** | **4** | **5** | **6** | **7** | **8** | **9** | **10** | **11** | **12** |  |
| Antunes 2016 [45] | Y | Y | N | CD | NR | Y | Y | N | Y | Y | Y | NA | **7 (63.6)** |
| Badr 2002 [46] | Y | Y | N | Y | Y | Y | Y | N | Y | Y | Y | NA | **9 (81.8)** |
| Baydur 2001 [35] | Y | Y | N | Y | NR | NR | Y | N | Y | Y | Y | NA | **7 (63.6)** |
| Ben-Dov 2009 [17] | Y | Y | N | Y | NR | NR | Y | N | Y | Y | NR | NA | **6 (54.5)** |
| Benedik 2009 [52] | Y | Y | N | CD | Y | Y | Y | N | Y | Y | Y | NA | **8 (72.7)** |
| Ceridon 2011 [18] | Y | Y | N | CD | NR | NR | Y | N | Y | Y | Y | NA | **6 (54.6)** |
| Chang 2005 [53] | Y | Y | N | CD | Y | Y | Y | N | Y | Y | Y | NA | **8 (72.7)** |
| Costa 2015 [54] | Y | Y | N | CD | Y | NR | Y | N | Y | Y | Y | NA | **7 (63.6)** |
| De 2012 [29] | Y | Y | N | Y | NR | NR | Y | N | Y | Y | Y | NA | **7 (63.6)** |
| Elkins 2005 [47] | Y | Y | N | Y | Y | Y | Y | N | Y | Y | Y | NA | **9 (81.8)** |
| Faggiano 1998 [58] | Y | Y | N | CD | NR | Y | Y | N | Y | Y | N | NA | **6 (54.5)** |
| Ganapathi 2015 [19] | Y | Y | N | CD | NR | NR | Y | N | Y | Y | Y | NA | **6 (54.5)** |
| Gianinis 2013 [48] | Y | Y | N | CD | NR | NR | Y | N | Y | Y | Y | NA | **6 (54.5)** |
| Kim 2012 [36] | Y | Y | N | CD | NR | NR | Y | N | Y | Y | Y | NA | **6 (54.5)** |
| Linn 2000 [33] | Y | Y | N | Y | NR | NR | Y | N | Y | Y | Y | NA | **7 (63.6)** |
| Manning 1999 [20] | Y | Y | N | CD | NR | Y | Y | N | Y | Y | Y | NA | **7 (63.6)** |
| McCoy 2010 [49] | Y | Y | N | Y | NR | NR | Y | N | Y | Y | Y | NA | **7 (63.6)** |
| Melam 2014 [30] | Y | Y | N | CD | NR | NR | Y | N | Y | Y | Y | NA | **6 (54.5)** |
| Meysman 1998 [3] | Y | Y | N | CD | NR | Y | Y | N | Y | Y | Y | NA | **7 (63.6)** |
| Miccinilli 2016 [40] | Y | Y | N | Y | Y | NR | Y | N | Y | Y | Y | NA | **8 (72.7)** |
| Mohammed 2017 [31] | Y | Y | N | CD | NR | NR | Y | N | Y | Y | Y | NA | **6 (54.5)** |
| Myint 2017 [42] | Y | Y | N | CD | NR | NR | Y | N | Y | Y | Y | NA | **6 (54.5)** |
| Naitoh 2014 [39] | Y | Y | N | CD | NR | Y | Y | N | Y | Y | Y | NA | **7 (63.6)** |
| Ogiwara 2002 [55] | Y | Y | N | CD | NR | Y | Y | N | Y | Y | Y | NA | **7 (63.6)** |
| Ottaviano 2016 [50] | Y | Y | N | CD | NR | NR | Y | N | Y | Y | Y | NA | **6 (54.5)** |
| Palmero 2005 [21] | Y | Y | N | CD | NR | Y | Y | N | Y | Y | N | NA | **6 (54.5)** |
| Park 2010 [34] | Y | Y | N | Y | NR | NR | Y | N | Y | Y | Y | NA | **7 (63.6)** |
| Patel 2015 [22] | Y | Y | N | CD | NR | NR | Y | N | Y | Y | Y | NA | **6 (54.5)** |
| Peces-Barba 2004 [56] | Y | Y | N | CD | NR | NR | Y | N | Y | Y | N | NA | **5 (45.5)** |
| Poussel 2014 [38] | Y | Y | N | Y | NR | NR | Y | N | Y | Y | Y | NA | **7 (63.6)** |
| Razi 2007 [32] | Y | Y | N | CD | NR | NR | Y | N | Y | Y | Y | NA | **6 (54.5)** |
| Roychowdhury 2011 [44] | Y | Y | N | CD | NR | NR | Y | N | Y | Y | NR | NA | **5 (45.5)** |
| Saxena 2006 [23] | Y | Y | N | CD | NR | NR | Y | N | Y | Y | NR | NA | **5 (45.5)** |
| Sebbane 2015 [41] | Y | Y | N | Y | Y | NR | Y | N | Y | Y | N | NA | **7 (63.6)** |
| Stewart 2000 [24] | Y | Y | N | CD | NR | Y | Y | N | Y | Y | N | NA | **6 (54.5)** |
| Terson de Paleville 2014 [37] | Y | Y | N | CD | NR | Y | Y | N | Y | Y | Y | NA | **7 (63.6)** |
| Terzano 2009 [57] | Y | Y | N | CD | NR | Y | Y | N | Y | Y | Y | NA | **7 (63.6)** |
| Tsubaki 2009 [28] | Y | Y | N | Y | NR | NR | Y | N | Y | Y | Y | NA | **7 (63.6)** |
| Varrato 2001 [25] | Y | Y | N | CD | NR | NR | Y | N | Y | Y | NR | NA | **5 (45.5)** |
| Vilke 2000 [26] | Y | Y | N | CD | NR | NR | Y | N | Y | Y | Y | NA | **6 (54.5)** |
| Wallace 2013 [51] | Y | Y | N | Y | NR | NR | Y | N | Y | Y | Y | NA | **7 (63.6)** |
| Watson 2005 [43] | Y | Y | N | CD | NR | NR | Y | N | Y | Y | N | NA | **5 (45.5)** |
| Yap 2000 [27] | Y | Y | N | CD | NR | Y | Y | N | Y | Y | Y | NA | **7 (63.6)** |

Abbreviations CD Cannot determine; NA Not applicable; NR Not reported; N No; Y Yes

^a^ Questions from the quality assessment tool, as presented by Kunstler et al [16]

1. Was the study question or objective clearly stated?
2. Were eligibility/selection criteria for the study population pre-specified and clearly described?
3. Were the participants in the study representative of those who would be eligible for the test/service/intervention in the general or clinical population of interest?
4. Were all eligible participants that met the pre-specified entry criteria enrolled?
5. Was the sample size sufficiently large to provide confidence in the findings?
6. Was the test/service/intervention clearly described and delivered consistently across the study population?
7. Were the outcome measures pre-specified, clearly defined, valid, reliable, and assessed consistently across all study participants?
8. Were the people assessing the outcomes blinded to the participants' exposures/interventions?
9. Was the loss to follow-up after baseline 20% or less? Were those lost to follow-up accounted for in the analysis?
10. Did the statistical methods examine changes in outcome measures from before to after the intervention? Were statistical tests done that provided p values for the pre-to-post changes?
11. Were outcome measures of interest taken multiple times before the intervention and multiple times after the intervention (i.e., did they use an interrupted time-series design)?
12. If the intervention was conducted at a group level (e.g., a whole hospital, a community, etc.) did the statistical analysis take into account the use of individual-level data to determine effects at the group level? (*If this question is not applicable, total score is out of 11, not 12.)
