# Supplementary material for: The effect of body position on pulmonary function: a systematic review
Source: BMC Pulm Med. 2018 Oct 11;18:159. doi: 10.1186/s12890-018-0723-4 (PMC6180369; doi:10.1186/s12890-018-0723-4)
Supplement: Supplementary file 2 — Table S2. Statistically significant differences in pulmonary function between the various body positions [3, 17–28, 30, 31, 33, 34, 37–41, 43–48, 50–54, 56]. (DOCX 104 kb) [file 12890_2018_723_MOESM2_ESM.docx]

**Additional file 2: Table S2 Statistically significant differences in pulmonary function between the various body positions**

| **Pulmonary Function** | **1^st^ author**  **(year)** | **Standing** | **Sitting** | | **Supine** | **RSL** | **LSL** | **P value** |
| --- | --- | --- | --- | --- | --- | --- | --- | --- |
| **FVC**  ***(L)*** | **Healthy** | | | | | | | |
|  | Ceridon (2011) [18] | N/A | 4.3±0.9  *98±16% predicted* | 4.0±1.0  *89±16% predicted)* | | N/A | N/A | P<0.05 |
|  | Ganapathi (2015) [19] | 3.71±0.34 | 3.50±0.42 | 3.28±0.43 | | 3.46±0.39 | 3.38±0.35 | P<0.04 |
|  |  | 3.71±0.34 | 3.50±0.42 | 3.28±0.43 | | 3.46±0.39 | 3.38±0.35 | P<0.04 |
|  |  | 3.71±0.34 | 3.50±0.42 | 3.28±0.43 | | 3.46±0.39 | 3.38±0.35 | P<0.009 |
|  | Manning (1999) [20] | N/A | 3.97±0.87 | N/A | | 3.87±0.89 | 3.84±0.85 | P<0.01 |
|  |  | N/A | 4±0.94 | N/A | | 3.87±0.89 | 3.84±0.85 | P<0.01 |
|  | Meysman (1998) [3] | N/A | 5.23±1.15 | 4.99±1.07 | | 4.87±1.08 | 4.99±1.09 | P<0.0001 |
|  | Patel (2015) [22] | 3.90 | 4.04 | 3.73 | | N/A | N/A | P<0.001 |
|  |  | 3.90 | 4.04 | 3.73 | | N/A | N/A | P<0.001 |
|  | Saxena (2016) [23] | Males 4.07±0.51  qs 2.89±0.41 | Males 3.99±0.52  Females 2.83±0.42 | Males 3.73±0.48  Females 2.64±0.39 | | N/A | N/A | P<0.05 |
|  |  | Males 4.07±0.51  Females 2.89±0.41 | Males 3.99±0.52  Females 2.83±0.42 | Males 3.73±0.48  Females 2.64±0.39 | | N/A | N/A | P<0.05  Males |
|  | Stewart (2000) [24] | N/A | Males  5.59±0.29 *Mean±SE* | Males  5.16±0.27 *Mean±SE* | | N/A | N/A | P<0.05 |
|  | Varrato (2001) [25] | N/A | 4.1  *101% predicted* | 3.8  *95% predicted* | | N/A | N/A | P<0.05 |
|  | Vilke (2000) [26] | N/A | Males  5.34  *102±4% predicted* | Males  4.96  *95±4%*  *predicted* | | N/A | N/A | P<0.05 |
|  | Yap (2000) [27] | N/A | 4.33±0.43 *92.8±6.8% predicted*  *Mean±SE* | 4.03±0.38 *86.6±5.8% predicted Mean±SE* | | N/A | N/A | P<0.01 |
|  | **Lung disease** | | | | | | | |
|  | Melam (2014) [30] | 2.5±0.6 | 2.3±0.6 | 2.0±0.5 | | 2.3±0.6 | 2.2±0.1 | P<0.05 |
|  | Mohammed (2017) [31] | 3.35±0.31 | 3.07±0.29 | 2.60±0.30 | | 2.82±0.27 | 2.82±0.27 | P<0.05 |
|  |  | 3.35±0.31 | 3.07±0.29 | 2.60±0.30 | | 2.82±0.27 | 2.82±0.27 | P<0.05 |
|  | **Cardiac disease** | | | | | | | |
|  | Ceridon (2011) [18] | N/A | 3.7±1.0  *86±11% predicted* | 3.3±1.0  *77±13% predicted* | | N/A | N/A | P<0.05 |
|  | Palermo (2005) [21] | N/A | 3.4±0.9 *89.8±22.3 % predicted* | 3.3±0.8 *87.5±21.6% predicted* | | 3.2±0.9 *84.5±22.2% predicted* | 3.2±0.8 *85.1±20.7% predicted* | P<0.05 |
|  | Yap (2000) [27] | N/A | 2.70±0.29 *70.0±2.7% predicted*  *Mean±SE* | 2.35±0.30 *60.5±2.2 %predicted* *Mean±SE* | | N/A | N/A | P<0.05 |
|  | **Spinal cord injury** | | | | | | | |
|  | Ben-Dov (2009) [17] | N/A | N/D | N/D | | N/A | N/A | Supine>sitting, Median 21% higher, range 11–29% P=0.0005 |
|  | Linn (2000) [33] | N/A | N/D | N/D | | N/A | N/A | High  tetraplegia supine>sitting, 16% higher  P<0.05 |
|  | Park (2010) [34] | N/A | 2.30±0.56  *48.3±10*  *% predicted* | 2.60±0.65 54.2±10.4% predicted | | N/A | N/A | P<0.01 |
|  | Terson de Paleville (2014) [37]  *(% predicted)* | N/A | 64.7±13.8 | 70.4±11.4 | | N/A | N/A | Complete  motor injury  P=0.007 |
|  |  | N/A | 59.3±8.1 | 65.9±7.3 | | N/A | N/A | Cervical  complete motor injury  P=0.033 |
|  |  | N/A | 93.7±17.4 | 90.6±18.2 | | N/A | N/A | Thoracic  Incomplete motor injury  P=0.022 |
|  | **Neuromuscular diseases** | | | | | | | |
|  | Park (2010) [34] | N/A | 1.37±0.60  *34.5±15.6% predicted* | 1.17±0.60 *29.2±14.9% predicted* | | N/A | N/A | P<0.01 |
|  | Poussel (2014)  [38]  *(% predicted)* | N/A | 73.5±19.7 | 67.3±22.3 | | N/A | N/A | P<0.0001 |
|  | Varrato (2001) [25] | N/A | 3.1  *81% predicted* | 2.7  *69% predicted* | | N/A | N/A | P<0.05 |
| **VC**  ***(L)*** | **Healthy** | | | | | | | |
|  | Naitoh (2014) [39] | N/A | 3.89±0.79  *100.8±17.0%* | 3.72±0.81  *95.5±15.3%* | | N/A | N/A | P<0.05 |
|  | Roychowdhury (2011) [44] | N/A | Females  2.83±0.38 | Females  3.01±0.42 | | N/A | N/A | P<0.05 |
|  | **Heart disease** | | | | | | | |
|  | Yap (2000) [27] | N/A | 2.60±0.62 *Mean±SE* | 2.40±0.24  *Mean±SE* | | N/A | N/A | P<0.05 |
|  | **Spinal cord injury** | | | | | | | |
|  | Miccinilli (2016) [40] | N/A | 2.19±0.93 | N/D | | N/A | N/A | P<0.0001  79% Increase |
| **FEV1**  ***(L/s)*** | **Healthy** | | | | | | | |
|  | Ceridon (2011) [18] | N/A | 3.5±0.8  *100±13% predicted* | 3.1±0.8  *90±12% predicted* | | N/A | N/A | P<0.05 |
|  | Ganapathi (2015) [19] | 3.04±0.33 | 2.70±0.33 | 2.51±0.38 | | 2.48±032 | 2.56±0.29 | P<0.001 |
|  |  | 3.04±0.33 | 2.70±0.33 | 2.51±0.38 | | 2.48±0.32 | 2.56±0.29 | P<0.001 |
|  |  | 3.04±0.33 | 2.70±0.33 | 2.51±0.38 | | 2.48±0.32 | 2.56±0.29 | P<0.001 |
|  |  | 3.04±0.33 | 2.70±0.33 | 2.51±0.38 | | 2.48±0.32 | 2.56±0.29 | P<0.002 |
|  | Manning (1999) [20] | N/A | 2.76±0.67 | N/A | | 2.63±0.67 | 2.59±0.65 | P<0.001 |
|  | Meysman (1998) [3] | N/A | 4.42±0.87 | 4.13±0.8 | | 4.13±0.8 | 4.15±0.79 | P<0.0001 |
|  | Naitoh (2014) [39] | N/A | 3.43±0.76 | 3.07±0.76 | | N/A | N/A | P<0.05 |
|  | Patel (2015) [22] | 3.48 | 3.60 | 3.26 | | N/A | N/A | P<0.001 |
|  |  | 3.48 | 3.60 | 3.26 | | N/A | N/A | P<0.001 |
|  | Saxena (2006) [23] | Males 3.39±0.42  Females 2.53±0.39 | Males 3.34±0.40  Females 2.50±0.40 | Males 3.08±0.39  Females 2.28±0.37 | | N/A | N/A | P<0.05 |
|  |  | Males 3.39±0.42  Females 2.53±0.39 | Males 3.34±0.40  Females 2.50±0.40 | Males 3.08±0.39  Females 2.28±0.37 | | N/A | N/A | P<0.05 |
|  | Vilke (2000) [26] | N/A | 4.27  *104±3% predicted* | 3.96  *96±3%*  *predicted* | | N/A | N/A | P<0.05 |
|  | Yap (2000) [27] | N/A | 3.27±0.28 *102.8±6.3% predicted*  *Mean±SE* | 3.07±0.26 *96.7±6.1% predicted Mean±SE* | | N/A | N/A | P<0.01 |
|  | **Lung disease** | | | | | | | |
|  | Melam (2014) [30] | 2.0±0.5 | 1.9±0.6 | 1.7±0.5 | | 1.8±0.5 | 1.8±0.5 | P<0.05 |
|  | Mohammed (2017) [31] | 2.31±0.68 | 1.98±0.28 | 1.52±0.23 | | 1.74±0.24 | 1.74±0.24 | P<0.05 |
|  |  | 2.31±0.68 | 1.98±0.28 | 1.52±0.23 | | 1.74±0.24 | 1.74±0.24 | P<0.05 |
|  | **Cardiac disease** | | | | | | | |
|  | Ceridon (2011) [18] | N/A | 2.8±0.9  *85±15% predicted* | 2.5±0.8  *74±10% predicted* | | N/A | N/A | P<0.05 |
|  | Palermo (2005) [21] | N/A | 2.5±0.5 *84.7±16.9% predicted* | 2.5±0.5  *83.1±15.6% predicted* | | 2.3±0.4 *76.3±13.0% predicted* | 2.4±0.5  *77.8±14.5% predicted* | P<0.05 |
|  | Yap (2000) [27] | N/A | 2.16±0.25 *76.4±2.6% predicted*  *Mean±SE* | 1.89±0.29 *66.1±4.9% predicted*  *Mean±SE* | | N/A | N/A | P<0.01 |
|  | **Spinal cord injury** | | | | | | | |
|  | Linn (2000) [33] | N/A | N/D | N/D | | N/A | N/A | Supine vs. sitting  10% increase  P<0.05 |
|  | Miccinilli (2016) [40] | N/A | 1.51±0.86 | N/D | | N/A | N/A | Supine vs. sitting  66% increase P<0.001 |
|  | Tereson De Paleville (2014) [37]  *(% predicted)* | N/A | 76.1±21.5 | 71.4±21.4 | | N/A | N/A | Incomplete motor injury  P=0.002 |
|  |  | N/A | 85.8±19.2 | 80.6±22.2 | | N/A | N/A | Incomplete thoracic motor injury  P=0.042 |
|  | **Neuromuscular diseases** | | | | | | | |
|  | Poussel (2014) [38]  *(% predicted)* | N/A | 73.0±20.0 | 64.1±18.3 | | N/A | N/A | P<0.0001 |
|  | **Obesity** | | | | | | | |
|  | Sebbane (2015) [41]  *(mean BMI 45)* | N/A | 2.69±0.63 *97±12% predicted* | 2.48±0.6  *90±14% predicted* | | N/A | N/A | P=0.02 |
|  | Sebbane (2015) [41]  *(mean BMI 31)* | N/A | 2.83±0.58  *105±14% predicted* | 2.67±0.54  *99±16% predicted* | | N/A | N/A | P=0.044 |
| **FEV1/**  **FVC%** | **Healthy** | | | | | | | |
|  | Ganapathi (2015) [19] | 81.93±3.57 | 77.12±4.45 | 76.40±5.87 | | 74.34±5.88 | 73.47±6.97 | P=0.03 |
|  |  | 81.93±3.57 | 77.12±4.45 | 76.40±5.87 | | 74.34±5.88 | 73.47±6.97 | P<0.002 |
|  |  | 81.93±3.57 | 77.12±4.45 | 76.40±5.87 | | 74.34±5.88 | 73.47±6.97 | P<0.001 |
|  |  | 81.93±3.57 | 77.12±4.45 | 76.40±5.87 | | 74.34±5.88 | 73.47±6.97 | P<0.001 |
|  |  | 81.93±3.57 | 77.12±4.45 | 76.40±5.87 | | 74.34±5.88 | 73.47±6.97 | P<0.006 |
|  | Saxena (2006) [23] | Male  83.1±3.6 | Males  83.3±3.8 | Males 82.2±3.8 | | N/A | N/A | P<0.05 |
|  | Tsubaki (2009) [28] | N/D | Females  92.0±6.3 | Females  86.6±5.8 | | N/D | N/D | P<0.05 |
| **PEF**  ***(L/min)*** | **Healthy** | | | | | | | |
|  | Antunes (2016) [45] | N/A | 481±115.1 | 453.2±114.3 | | N/A | N/A | P<0.05 |
|  | Badr (2002) [46]  *(in ml/s)* | 571±24  *Mean±SE* | N/D | N/D | | N/D | N/A | P<0.04 |
|  | Gianinis (2013) [48] | N/A | Males 558±116.7  Females 404.7±45.1 | Males 530±118.2  Females 375±37.4 | | Males 526.7±117  Females 382.3±39.7 | Males 545.3±132.2  Females 393±38.7 | P<0.05 for  each group,  and for total  population |
|  | Meysman (1998) [3]  *(in L/sec)* | N/A | 10.02±2.99 | 9.32±2.22 | | 8.98±2.08 | 9.19±2.18 | P<0.0001 |
|  | Ottaviano (2016) [50] | N/D | N/D | N/A | | N/A | N/A | P=0.009 |
|  | Patel (2015) [22]  *(in L/sec)* | 7.78 | 8.33 | 7.35 | | N/A | N/A | P<0.001 |
|  |  | 7.78 | 8.33 | 7.35 | | N/A | N/A | P<0.001 |
|  | Stewart (2000) [24]  *(L/sec)* | N/A | 10.35±0.58 *Mean±SE* | 9.34±0.59 *Mean±SE* | | N/A | N/A | P<0.05 |
|  | Saxena (2006) [23] | Males 533.6±77.9  Females  350.5±66.4 | Males 520.4±77.5  Females 332.6±69.9 | Males 479.2±76.7  Females 305.9±62.0 | | N/A | N/A | P<0.05 |
|  |  | Males 533.6±77.9  Females  350.5±66.4 | Males 520.4±77.5  Females 332.6±69.9 | Males 479.2±76.7  Females 305.9±62.0 | | N/A | N/A | P<0.05 |
|  | Wallace (2013) [51] | Males  669±42  Females  462±42 | Males  615±42  Females 447±42 | N/A | | N/A | N/A | Males  P<0.001  Females P<0.05 |
|  | **Lung disease** | | | | | | | |
|  | Badr (2002) [46] | 284±40 | N/D | N/D | | N/D | N/A | P<0.01 |
|  | Elkins (2005) [47]  *(in L/sec)* | 6.35 | N/D | 5.79 | | N/D | N/A | P<0.05 |
|  | Mohammed (2017) [31] | 351.9±35.1 | 334.2±35.4 | 284.3±25.3 | | 310.4±28.6 | 310.4 ±28.6 | P<0.05 |
|  | **Spinal cord injury** | | | | | | | |
|  | Linn (2000) [33] | N/A | N/D | N/D | | N/A | N/A | Supine vs. sitting  12% increase  P<0.05 |
| **FRC**  ***(L)*** | **Healthy** | | | | | | | |
|  | Chang (2005) [53] | Males  3.26±0.83 | N/A | Males  2.34±0.49 | | N/A | N/A | P<0.001 |
|  | Yap (2000) [27] | N/A | 3.71±0.27  *Mean ±SE* | 3.05±0.24  *Mean±SE* | | N/A | N/A | P<0.01 |
|  | Watson (2005) [43] | N/A | 3.43±0.2  *Mean±SE* | 2.69±0.2 *Mean±SE* | | N/A | N/A | P<0.0001 |
|  | **Obesity** | | | | | | | |
|  | Benedik (2009) [52] | N/A | 2.13±0.56 | 1.65±0.43 | | N/A | N/A | P<0.001 |
|  | Sebbane (2015)  [41]  *(mean BMI 31)* | N/A | 2.5±0.3  *89±14% predicted* | 1.9±0.3  *68±13%*  *predicted* | | N/A | N/A | P=0.002 |
| **PEmax *(cmH2O)*** | **Healthy** | | | | | | | |
|  | Badr (2002) [46] | 143±10  *Mean±SE* | 133±10  *Mean±SE* | N/D | | N/D | N/A | P<0.001 |
|  |  | 143±10  *Mean±SE* | 133±10  *Mean±SE* | N/D | | N/D | N/A | P<0.04 |
|  | Costa (2015) [54] | N/A | 125.4±34.2 | 115.8±29 | | N/A | N/A | P<0.05 |
|  | **Lung disease** | | | | | | | |
|  | Badr (2002) [46] | 134±18 | N/D | N/D | | N/D | N/A | P<0.01 |
|  |  | 134±18 | N/D | N/D | | N/D | N/A | P<0.05 |
|  | Elkins (2005) [47] | 142 | N/D | N/D | | N/D | N/A | P<0.05 |
|  |  | 142 | N/D | N/D | | N/D | N/A | P<0.05 |
|  | **Spinal cord injury** | | | | | | | |
|  | Terson de Paleville (2014) [37] | N/A | 60.5±26.6 | 53.4±25.5 | | N/A | N/A | All subjects P=0.011 |
|  |  | N/A | 49.1±22.7 | 39.9±14.4 | | N/A | N/A | Complete  motor injury  P=0.031 |
|  |  | N/A | 64.0±28.6 | 56.7±22.2 | | N/A | N/A | Cervical incomplete  motor injury  P=0.040 |
| **PImax *(cmH2O)*** | **Healthy** | | | | | | | |
|  | Costa (2015) [54] | N/A | -92.8±20.05 | -84.1±15.1 | | N/A | N/A | P<0.05 |
|  | Meysman (1998) [3] | N/A | -106±27.7 | -97.2±25.6 | | -99.6±29.6 | -99.6±31.4 | P<0.0001 |
|  | **Spinal cord injury** | | | | | | | |
|  | Terson de Paleville (2014) [37] | N/A | -93.1±19.6 | -77.2±18.0 | | N/A | N/A | Thoracic  complete  motor injury P=0.041 |
| **DLCO *(ml/mmHg/min)*** | **Healthy** | | | | | | | |
|  | Ceridon (2011) [18] | N/A | 17.4±5.6 | 15.7±4.6 | | N/A | N/A | P<0.05 |
|  | Peces-Barba (2004) [56] | N/A | 29.0±1.9  *Mean±SE* | 31.6±2.3  *Mean±SE* | | N/A | N/A | P<0.001 |
|  | Palermo (2005) [21] | N/A | N/D | N/D | | N/D | N/D | P<0.02 |
|  | Stewart (2000) [24]  *(ml/mmHg/min/l)* | N/A | 4.66±0.15 | 5.22±0.13 | | N/A | N/A | P=0.022 |
|  | **Cardiac disease** | | | | | | | |
|  | Palermo (2005) [21] | N/A | N/D | N/D | | N/D | N/D | P<0.02 |

All units are as mentioned in left hand bar unless otherwise mentioned next to author name.

All lung function and pressure values are mentioned in the grey and white boxes unless they were not reported in the study text

Values are mean±S.D. unless specified others.

N/D No data – parameter was measured but exact values were not reported in the study

N/A –Not Available – not evaluated in the study

|  | Higher value |
| --- | --- |
|  | Lower value |

*BMI, body mass index; DLCO, diffusing capacity of the lungs for carbon monoxide, FEV1, forced expiratory flow in 1 second; FRC, functional residual capacity; FVC, forced vital capacity; LSL, left side lying; PEF, peak expiratory flow; PEmax, maximal expiratory pressure; PImax, maximal inspiratory pressure; RSL, right side lying; VC, vital capacity*
